# A Brief Cognitive Behavioural Intervention for Parents of Anxious Children: Feasibility and Acceptability Study

**DOI:** 10.1007/s10566-022-09704-x

**Published:** 2022-08-12

**Authors:** C Jewell, A Wittkowski, S Collinge, Daniel Pratt

**Affiliations:** 1 Centre for New Treatments and Understanding in Mental Health, School of Health Sciences, The University of Manchester, Manchester, UK; 2 Greater Manchester Mental Health NHS Foundation Trust, Manchester, UK; 3 Manchester Academic Health Science Centre, Manchester, UK; 4 Lancashire Care NHS Foundation Trust, Preston, UK; 5 Division of Psychology & Mental Health, University of Manchester, 2nd Floor Zochonis Building, M13 9PL, 0161 306 0400 Manchester, United Kingdom

**Keywords:** Parent-only, Non-controlled trial, Child anxiety, Cognitive behavioural therapy, Treatment

## Abstract

**Background:**

Parent-only psychological interventions can be effective treatments for child anxiety. Involving parents in treatment may be beneficial for children, ensuring that interventions are delivered effectively in a supportive environment. Few studies have investigated the feasibility and acceptability of parent-only interventions for child anxiety.

**Objective:**

In this study, we report on feasibility, acceptability and preliminary clinical outcomes of a brief cognitive behavioural group intervention for parents of children (4- to 10-years-olds) experiencing anxiety in the absence of a diagnosed anxiety disorder.

**Method:**

Parent participants attended a three-session group intervention delivered online. We collected feasibility information (recruitment and retention rates); parents and children (when appropriate) completed acceptability and clinical outcome measures after each session. Participants were also interviewed about the acceptability of the intervention and study processes.

**Results:**

Nineteen parents consented to take part (child mean age 6.47, SD 1.23). Participant retention rates (68.4%) and intervention satisfaction (total mean CSQ score 28.52) were high​. Calculated effect sizes were moderate to large for parent-rated outcomes, small for child self-reported anxiety, and small to moderate for parent confidence/efficacy. Thematic analysis of interview data identified benefits, such as connecting with parents and learning strategies, as well as challenges associated with the intervention.

**Conclusions:**

Attendance appeared to be associated with positive changes for parents and children. Overall, participants found this to be an acceptable and useful intervention. These findings demonstrated the potential benefit of a brief intervention for parents of anxious children. A larger trial is required to further investigate these preliminary findings.

## Introduction

Children and young people are increasingly affected by mental health problems, with 8% of 5- to 19-year-olds in the United Kingdom (UK) experiencing an emotional disorder, such as anxiety, depression or bipolar disorder (NHS Digital, 2017). Anxiety disorders are the most commonly reported, affecting 3.9% of 5- to 10-year-olds and 7.9% of 11- to 16-year-olds in the UK (NHS Digital, 2017). However, the true prevalence of anxiety problems is likely to be much higher since these figures fail to include children and young people who do not access mental health services.

Anxiety disorders are typically characterised by excessive, persistent feelings of fear and worry which create significant distress and have a negative impact on daily functioning (American Psychiatric Association, 2013). The Diagnostic and Statistical Manual of Mental Disorders, Fifth Edition (DSM–5) (American Psychiatric Association, 2013) includes panic disorder, agoraphobia, generalised anxiety disorder, specific phobia, social anxiety disorder, separation anxiety disorder and selective mutism within the criteria for anxiety disorders. Anxiety disorders can have a negative impact on children’s academic ability, social functioning and general family life (Nail et al., 2015; Settipani & Kendall, 2013; Towe-Goodman et al., 2014) and have been associated with later development of mental health problems (Bittner et al., 2007).

Cognitive Behavioural Therapy (CBT) is an effective treatment for childhood anxiety (James et al., 2015). However, due to increasing demands on psychology services, CBT is not widely available to all who may need it, particularly children without a diagnosis of anxiety disorder, whose symptoms of anxiety may not meet the threshold for accessing Child and Adolescent Mental Health Services (CAMHS), or whose parents may be reluctant or unable to access CAMHS. Therefore, alternative interventions to child-focused CBT may also be needed.

Children typically rely on parents to seek help on their behalf, yet parents report barriers to accessing psychological help for their child. One of the most commonly reported barriers is perceived social stigma and perceived negative attitudes from others (Reardon et al., 2017). Some parents believe their children are more likely to experience and be impacted negatively by stigma, suggesting that parents may prefer to attend an intervention which does not directly involve their child (Dempster et al., 2013).

A feasible alternative to child-focused CBT may be brief, low intensity interventions exclusively for parents. This approach is consistent with National Institute for Health and Care Excellence guidelines (NICE, 2013); family involvement in the treatment of child anxiety can create a supportive environment and ensure that interventions are delivered effectively, particularly with young children.

Low intensity interventions are typically brief in nature and can be delivered in groups, making them less costly in terms of time and resources. As well as potential cost-effectiveness, group interventions can help reduce feelings of isolation as participants hear other’s experiences of similar difficulties, providing the opportunity for peer support and connection (Yalom & Leszcz, 2005). Low intensity interventions may be particularly well-suited to parents because longer, more intensive interventions would place a high burden on parents who are likely to have other family commitments and priorities (Tully & Hunt, 2016). Brief interventions could therefore improve accessibility and facilitate parent engagement with psychological support for their child.

Parent-only interventions can be effective treatments for child anxiety (see review by Jewell et al., 2022). Typically, these interventions involve attending face-to-face sessions delivered in a clinical setting, or accessing self-help materials with or without additional support from a therapist. In a meta-analysis of six RCTs investigating the efficacy and acceptability parent interventions for anxiety disorders in children and adolescents (Yin et al., 2021), parent-only CBT was significantly more effective than waitlist control for reducing child anxiety symptoms (*g* =-0.72, 95% -1.41-0.03, p = 0.04). However, acceptability of parent-only CBT was not significantly different to waitlist control (RR 0.92, 95% CI 0.53–1.62, p = 0.77) and was significantly worse than parent and child CBT (RR 1.93, 95% CI 1.05–3.57, p = 0.03). Two studies included in the meta-analysis focused on younger children under the age of 9 years old (Monga et al., 2015; Cartwright-Hatton et al., 2011; Yin et al., 2021) found that attrition was higher for parent-only CBT compared to CBT involving both parents and children.

Very few other studies have specifically investigated the feasibility and acceptability of parent-only interventions for child anxiety (Chavira et al., 2014, 2018; Creswell et al., 2010; Lebowitz et al., 2014). These studies demonstrated that interventions were feasible and acceptable to parents, dropout rates were low and parents reported high satisfaction as well as improvements in child anxiety symptoms. However, each of these studies identified eligible children as meeting diagnostic criteria for anxiety disorders and only one previous study focused specifically upon children under the age of 12 years old (Creswell et al., 2010).

As such, there is a gap in the literature regarding brief interventions delivered as an early intervention for parents of children experiencing mild to moderate anxiety, in the absence of a diagnosed anxiety disorder. Interventions for children experiencing anxiety are important in order to prevent problems escalating into adolescence and adulthood. Interventions involving parents may be particularly valuable for preschool and school-aged children due to the significant influence of parents on child development and the opportunities for parents to utilise anxiety management strategies consistently through routine activities and interactions (Mahoney & Wiggers, 2007).

There is a need for studies to explore feasibility and acceptability during development and refinement of any intervention (Shahsavari et al., 2020); this process may be particularly important with interventions for parents in relation to the reported barriers to accessing support and high demands for busy parents. The purpose of the present study was to investigate the feasibility and acceptability of a brief, cognitive behavioural group intervention for parents of children experiencing symptoms of anxiety. The intervention was offered in community settings with the intention of increasing accessibility for parents who may be unwilling or unable to access CAMHS, as well as reducing any potential stigma for both parents and children. The aims of the study were:

1. to examine the feasibility of recruiting, engaging and retaining parents of children experiencing symptoms of anxiety in a brief cognitive behavioural group intervention,
2. to investigate parents’ views on the acceptability of the intervention and outcome measures and.
3. to explore the potential clinical benefits associated with receiving the intervention in terms of anxiety symptoms in children reported by parent participants.

## Method

### Design and Ethical Approval

This study used an uncontrolled pre-post design to investigate the feasibility and acceptability of a brief cognitive behavioural group intervention. Ethical approval was obtained from the University Research Ethics Committee (Ref: 2020-7825-12919). All participants were offered written information on the study prior to taking part and provided written, informed consent. The authors declare no conflict of interest.

### Inclusion Criteria

Inclusion criteria were parents of primary school children aged between 4 and 10 years old, who reported their child to be experiencing symptoms of anxiety, in the absence of a pre-existing diagnosis of an anxiety disorder. Anxiety was indicated by T-scores of 60 or above on the Preschool Anxiety Scale (Spence et al., 2001) for parents of children aged 4 to 6 years, or the Spence Children’s Anxiety Scale – Parent version (SCAS-parent; Spence 1998) for parents of children aged 7 to 10 years. T-scores of 60 represent subclinical or elevated levels of anxiety (Spence, 1998). Screening questionnaires were used in line with the aim of increasing access to psychological support for parents of anxious children without a diagnosed anxiety disorder, who may find it difficult to access services.

Due to the nature of the intervention, proficiency in English was required and parents with a pre-existing diagnosis of intellectual disability were excluded (determined by self-report screening questionnaire). Parents whose children had a prior diagnosis of Autism Spectrum Disorder were excluded from the study. It was also necessary for parents to have the use of an electronic device and access to the internet in order to participate.

### Recruitment

Participants were recruited between 3 March and 10 December 2020. Two schools which differed in terms of their size, location and socio-economic status of pupils were identified to support recruitment. Due to school closures in response to the Covid-19 pandemic, which began in March 2020, only one school continued to support recruitment between March and September 2020. Other primary schools, children’s centres, out of school clubs, children’s sports clubs, girl guides and scouting groups were contacted between July and September 2020. Study information, including a poster, participant information sheet, recruitment email and social media text, was shared with potential participants via existing structures within the organisation (e.g., newsletter, email, social media, blog post). Participants self-selected into the study.

### Feasibility Outcomes

**Feasibility Statistics.** Feasibility was determined according to recruitment data (participants expressing interest, eligibility, rate of recruitment) and participant retention (number of sessions attended, number of participants who did not complete the study).

**Client Satisfaction.** Participants were asked to complete the 8-item Client Satisfaction Questionnaire-8 (CSQ-8; Larsen et al., 1979) which is widely used across mental health services to assess satisfaction with care and support. Responses are based on a scale from 1 to 4, with higher scores indicating higher satisfaction. Total scores range from a minimum of 8 to a maximum of 32. The CSQ-8 is highly reliable, valid and standardised (Attkisson & Zwick, 1982). For this study, Cronbach’s alpha was 0.77.

### Clinical Outcomes

**The Preschool Anxiety Scale (PAS;** Spence et al., 2001). The PAS is a questionnaire, completed by the parent, identifying anxiety symptoms in children aged 2.5 to 6.5 years old. The 28 items provide an overall measure of anxiety representing five anxiety subtypes: generalised anxiety disorder, social phobia, obsessive compulsive disorder, physical injury fears and separation anxiety. Responses are based on a 4-point scale from 0 to 3. Only the total score was used in the present study; higher scores indicate higher anxiety, with scores ranging from 0 to 112. The PAS has been found to have good construct validity, internal consistency and reliability (Spence et al., 2001).

**The Spence Children’s Anxiety Scale–Parent (SCAS-parent;** Spence 1998**).** The 38-item SCAS provides both a total anxiety score and six subscale scores. In addition to the five subtypes captured by the PAS, ‘panic attack and agoraphobia’ is included. Only the total score was used in the present study. Scores range from a minimum of zero to a maximum possible score of 114. The parent version of the SCAS has good reliability and validity (Nauta et al., 2004). Cronbach’s alpha for this study was 0.70.

**The Spence Children’s Anxiety Scale (SCAS-child;** Spence 1998**).** The SCAS is a child self-report measure which also aims to assess symptoms relating to six subtypes of anxiety. It consists of 44 items, mirroring the SCAS–parent version with the exception of six positive filler items (e.g., “I am good at sports”). Cronbach’s alpha for this study was 0.71.

**The Child Adjustment and Parent Efficacy Scale (CAPES;** Morawska et al., 2014). The CAPES is a self-report questionnaire identifying parents’ self-efficacy in managing their child’s behavioural and emotional difficulties. Parents’ beliefs about their ability to parent effectively have been linked to child functioning and outcomes (Jones & Prinz, 2005). Higher scores indicate greater levels of emotional or behavioural difficulties in children. Separate scores are available for the four subscales of behaviour, emotion, intensity and efficacy. Scores for behavioural problems range from 0 to 72 and emotional problems scores range from 0 to 9. The intensity score (sum of emotional and behavioural problems) has a maximum score of 81. The parent efficacy score represents the total of parent confidence ratings in managing emotional and behavioural difficulties with scores ranging from 19 to 190 and higher scores indicating greater levels of parent efficacy. A systematic review of 34 outcome measures suggested the use of the CAPES with parents of school aged children due to the quality of its psychometric properties (Wittkowski et al., 2017). The CAPES has been found to have good internal consistency and validity (Morawska et al., 2014). In this study, Cronbach’s alpha for intensity scores was 0.73 and efficacy scores was 0.84.

Permission was sought and granted from the authors of the PAS, SCAS and CAPES to develop an online survey format of the questionnaire measures, using the Select Survey application at the University of Manchester.

### Qualitative Outcomes

Participants were asked to complete a brief, bespoke, written evaluation questionnaire on completion of the intervention. All participants were offered the opportunity to participate in a semi-structured interview, conducted by the first author (CJ) who also delivered the intervention. The aim of the interview and evaluation form was to gather preliminary data regarding the intervention’s acceptability, its delivery format and the suitability of the outcome measures. As such, the content of the interview topic guide and evaluation form was informed by literature on the feasibility of interventions and evaluation of parent interventions, discussions within the research team, and previous experiences of evaluating interventions. Participants were asked to identify any barriers/difficulties to attending and engaging in the intervention, as well as any future recommendations.

### Procedure

Interested parents were asked to contact the research team directly and were then provided with further information about the study. If they decided to take part, participants were assigned a unique identifier code and their eligibility was assessed. Participants were asked to complete a consent form, a family background questionnaire for demographic information and the PAS or SCAS-parent version as a screening tool depending on the child’s age. All participants received an email indicating their eligibility status for the study; those who were not eligible were provided with recommendations for self-help books (for example “Overcoming your child’s fears and worries: A self-help guide using cognitive behavioural techniques” by Cathy Creswell and Lucy Willetts) and information for further support (for example Place2Be, NHS Direct, Mind). Eligible participants received an email invitation to attend the intervention, delivered via the online videoconferencing platform Zoom. Participants were allocated to intervention groups on a first come first served basis, regardless of demographics such as child age, school, parent/child gender. The intervention was delivered after a recruitment period of six weeks with the aim of maximising participant retention to the study. Recruitment to the study continued until a sufficient number of participants had completed the intervention to provide a meaningful analysis of the data, in line with sample sizes from previous feasibility studies investigating interventions for child anxiety (Comer et al., 2012; Jolstedt et al., 2018) because the primary aim of the study was to assess feasibility and acceptability. Blinding of study procedures was not accomplished.

The intervention was offered at different times (for example, morning and evening), to facilitate engagement. Intervention dates were fixed prior to recruitment to prioritise the certainty of intervention delivery. To reduce attrition, delivery of the intervention was not dependent on participant numbers; as such, the intervention went ahead as planned if at least one participant had been recruited.

Parent participants were asked to complete either the PAS or SCAS-parent version, CAPES and CSQ-8 after each intervention session. Parents of children aged 7 years old and over were also asked to encourage their child to complete the SCAS-child questionnaire independently after each intervention session, offering support when required. At the end of the intervention, participants were asked to complete an evaluation questionnaire. Email reminders were sent by the researcher to the participants between sessions to encourage completion of questionnaires. Following completion of the intervention, participants were offered a £10 e-voucher thanking them for their time. Participants were also debriefed and provided with contact details for local services, should they need further support.

All participants were invited to attend an interview within four weeks of completing the intervention to gain more detailed information regarding acceptability. Interviews were led by a topic guide. Participants received written information about this aspect of the study and were asked to provide additional written confirmation of consent to be interviewed.

### Intervention

The brief cognitive behavioural group intervention, developed by SC, was designed to be delivered over three sessions, totalling 5½ hours. It was decided that the intervention should be brief to maximise parent engagement and improve cost-effectiveness in line with NHS focus on providing efficient, high quality care (NHS, 2017). Sessions were delivered fortnightly over a six-week-period.

The development of the intervention was informed by NICE guidelines which identify CBT as the primary therapeutic intervention and recommend working collaboratively with parents in the treatment of child anxiety (NICE, 2013). The content of the intervention was influenced by existing CBT interventions for parents of children experiencing anxiety (Cartwright-Hatton et al., 2010; Creswell & Willetts, 2007) as well as literature on attachment and parenting (Golding & Hughes, 2012). A range of strategies were covered in the intervention to support parents of children ranging from ages 4 to 10 years; for example, play-based strategies for younger children and creating a thought detective to challenge thoughts for older children. Examples of adapting strategies for children of different ages were provided to parents during intervention sessions.

It was initially planned to deliver the intervention in primary schools. Although there is limited evidence for parent-only interventions delivered in educational settings, the school environment was hypothesised to be a community setting where parents might feel more able to access support without perceived social stigma. However, the outbreak of COVID-19 and subsequent national lockdown across the UK in March 2020 removed the option of a face-to-face group. It was therefore decided to deliver the intervention online via a videoconferencing platform, consistent with the aim of improving accessibility of psychological support to parents. All groups were delivered by the same trained facilitator (CJ) who received training and supervision by SC. A fidelity checklist was developed to record treatment adherence. The structure and content of the intervention is described in Table 1.

**Table 1:** Structure and Content of the Intervention

Session	Content	Homework Tasks	Duration
Session 1: What is anxiety	Introduction/psychoeducation: • Discuss parents’ aims, hopes and expectations • Introduction to anxiety • Explain fight or flight • Discuss chimp brain analogy • Discuss importance of meeting with parents using ‘anxiety cake’ analogy • Group activity - discuss signs, symptoms and triggers of anxiety in children • Discussion of what parents already do that helps CBT Formulation: • Discuss thoughts, feelings, behaviour cycle as a group using parent example Strategies: • Creating physical security (routine, boundaries, consistency) • Creating emotional security (playfulness, acceptance, curiosity and empathy; PACE) • Managing emotions (bubbles/balloons, newspaper punch, relax like a cat)	• Draw out basic CBT formulation for own child Practice strategies discussed during the session: • Creating physical security with routine, boundaries and consistency • Hearing worry with playfulness, acceptance, curiosity and empathy • Managing emotion with bubbles, balloons, newspaper punch and relax like a cat	2 h
Session 2: Building on formulation and developing strategies	Introduction/psychoeducation • Recap of previous session and review of homework • Avoidance and maintenance of anxiety CBT formulation: • Discuss systemic CBT formulation as a group using parent example • Impact on parents and importance of self-care Strategies: • Praise and rewards • Spotting anxious thoughts – talking to your child about anxiety • Evaluating thoughts using the worry tree • Problem solving • Thought challenging • Exposure to difficult situations • Discuss child’s motivation to change	Consider systemic CBT formulation Engage in own self-care activities Practice strategies discussed during session: • Praise and rewards • Spot warning signs and ask your child about their worries • Practice using the worry tree • Use 4-step problem solving • Evaluate anxious thoughts by weighing up the evidence • Help your child test out their fears by designing experiments or exposure to the situation	2 h
Session 3: Review	Recap of previous session and review of homework Group discussion troubleshooting any difficulties implementing strategies Reflecting on positive changes	Continue to practice strategies from sessions 1 and 2	1.5 h

### Data Analysis

Descriptive statistical analysis was used to present information regarding the feasibility of intervention delivery and study procedures. Frequency statistics and percentages were calculated to establish recruitment rates, attendance and retention rates. Mean scores and standard deviations on the CSQ-8 were also reported.

Means and standard deviations were calculated for clinical outcome data. Pre-test post-test effect sizes (Cohen’s *d*) were then computed (Morris, 2008) to provide preliminary information regarding any changes associated with receiving the intervention. Typically, d = 0.2 represents a small effect, d = 0.5 indicates a medium effect and d = 0.8 represents a large effect size. Post-intervention data were analysed using the intention-to-treat principle; any missing data were imputed using last observation carried forward. Individual participant’s pre- and post-scores were assessed for clinically significant change using the reliable change index (RCI); an RCI greater than 1.96 is indicative of clinically significant change (Jacobson & Truax, 1991).

Qualitative interviews were transcribed verbatim; transcripts and audio-recordings were then cross-checked for accuracy by the first author (CJ). Written evaluation forms and qualitative interview transcripts were analysed using thematic analysis (Braun & Clarke, 2006). NVivo 12 qualitative data analysis software was used to facilitate coding (QSR International Pty Ltd., 2018). Familiarisation with the data began with the transcription itself; transcripts were then repeatedly read and any relevant thoughts and observations were noted. Initial codes identifying potential features, patterns and themes in the data were produced. These codes were then collated based on similarities or overlapping concepts, from which initial themes and subthemes were developed. Themes were then reviewed and refined in relation to the coded data. Broader main themes were developed and refined from the initial themes. All themes were agreed by the research team. Data saturation was established when no new relevant information emerged from the interviews and analysis, and no new themes were identified. Evidence suggests that a total of 12 interviews is a sufficient sample size to reach data saturation in thematic analysis (Ando et al., 2014).

### Reflexivity Statement

All authors were psychologists with previous experience of working with parents and families which is likely to have informed their assumptions and biases. The first author (CJ) facilitated the intervention and conducted and transcribed all interviews. This approach resulted in full immersion with the data; however, the potential for introducing bias is acknowledged. Participants were aware that the first author was a trainee clinical psychologist acting in the role of a researcher, which may have impacted their expectations and assumptions throughout the research process.

## Results

### Participant Characteristics

A total of 35 parents expressed an interest in participating in the study. Seven parents did not meet inclusion criteria because their children had additional needs (n = 4), were outside of the study’s age range (n = 2) or presented with low levels of child anxiety (i.e., T-score < 60; n = 1). Three parents declined to participate and six parents did not complete eligibility screening questionnaires. Nineteen parents consented to take part and were assigned to the intervention, i.e., 54.3% of those expressing an interest in the study were converted into study participants.

Participant characteristics are described in Table 2. The sample consisted of 16 mothers and three fathers. Almost all participants self-identified as White British; one participant identified as Irish. The majority of participants were between 35 and 44 years of age (n = 11, 57.9%) and were married or in a civil partnership (n = 11, 57.9%). Children were 11 boys and eight girls from ten different schools in Greater Manchester and East Lancashire, with ages ranging from 4 to 9 years.

**Table 2:** Participant Characteristics

		N	%
Age	25–34 35–44 45–54 Missing	5 11 1 2	26.3 57.9 5.3 10.5
Gender	Female Male	16 3	84.2 15.8
Ethnicity	White British Irish	18 1	94.7 5.3
Child’s age	4 5 6 7 8 9 10 Child’s age M (SD)	1 3 5 8 0 2 0 6.47 (1.23)	5.3 15.8 26.3 42.1 0 10.5 0
Child’s gender (reported by parent)	Female Male	8 11	42.1 57.9
Relationship status	Single In a relationship/co-habiting Married/Civil Partnership Separated Divorced	1 3 11 3 1	5.3 15.8 57.9 15.8 5.3
Employment status	Full time Part time Student Unemployed Retired Missing	6 6 1 3 1 2	31.6 31.6 5.3 15.8 5.3 10.5
Previously offered/attended courses related to child’s wellbeing	Yes No	6 13	31.6 68.4

### Feasibility Outcomes

**Fig. 1:**
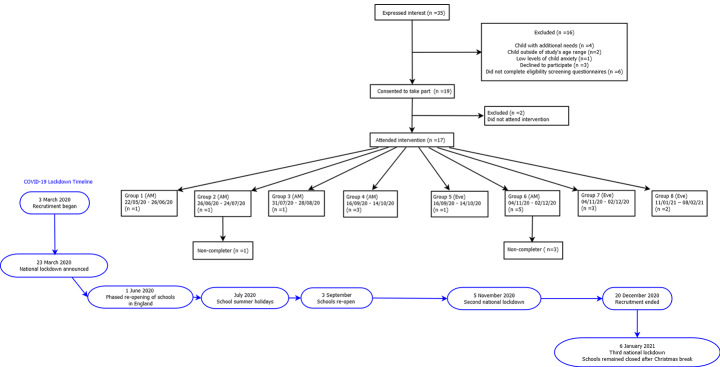
Flowchart of Participants Through the Study

**Recruitment.** Nineteen of 35 parents (54.3%) expressing interest in the study consented to participate and 17 (89.5%) of those consenting actually took part. Participants were invited to attend one of eight groups delivered between March 2020 and February 2021. Four of the groups were delivered with just one participant (see Fig. 1 for participant flow through the study). All 17 participants attending at least one intervention session were offered an interview after the intervention and 12 participants (70.6%) agreed to take part.

**Retention.** Frequencies and percentages for attendance and attrition are described in Table 3. Two participants did not attend any intervention sessions due to competing demands. Four participants only attended one session and as a result did not complete the intervention. Of these four participants, two reported work commitments as a reason for non-attendance and two participants did not provide reasons when contacted. Participants appeared engaged during the sessions, contributing during exercises and discussions, reflecting on homework tasks and sharing ideas., 11 participants (57.9%) completed all questionnaire measures. The retention rates of approximately 70% for intervention completion and almost 60% for questionnaire completion were considered to be good.

**Table 3:** Retention Rates

		N	%
Overall attendance	Session 1	16/19	84.3
	Session 2	14/19	73.7
	Session 3	13/19	68.4
Attended 3/3 sessions		13/19	68.4
Non-completers (attended fewer than 2 sessions)		6/19	31.6

**Treatment Fidelity.** All intervention components were successfully delivered in all sessions across the eight intervention groups, as measured by the treatment fidelity checklist.

**Client Satisfaction Questionnaire.** Mean CSQ-8 scores across the intervention are presented in Table 4. Session three obtained the highest satisfaction rating overall. The lowest scoring item was obtained in the first session for the question “to what extent has our service met your needs?” (2.93; SD = 0.73). The highest scoring items involved the quality of the service, overall satisfaction and recommending the service to a friend. Overall, scores on the CSQ-8 suggest that participants found the intervention to be acceptable.

**Table 4:** Mean Scores on CSQ-8

Item	Mean (SD)
1. How would you rate the quality of the service you received?	3.72 (0.46)
2. Did you get the kind of service you wanted?	3.61 (0.58)
3. To what extent has our service met your needs?	3.30 (0.76)
4. If a friend were in need of similar help, would you recommend our service to him or her?	3.70 (0.55)
5. How satisfied are you with the amount of help you received?	3.59 (0.54)
6. Have the services you received helped you to deal more effectively with your problems?	3.33 (0.52)
7. In an overall, general sense, how satisfied are you with the services you received?	3.70 (0.51)
8. If you were to seek help again, would you come back to our service?	3.59 (0.59)
Total mean score (out of a maximum of 32)	28.52 (3.71)
*Session 1 n = 14, Session 2 n = 16, Session 3 n = 16*	

### Qualitative Outcomes

**Fig. 2:**
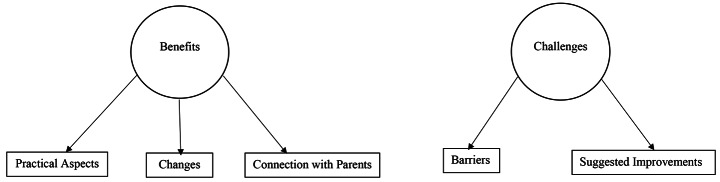
Diagrammatic Representation of Themes

Two main themes and five sub-themes were identified following analysis of 12 interviews, highlighting benefits as well as challenges associated with the intervention (Fig. 2).

## Theme 1: Benefits Associated with the Intervention

***Practical Aspects.*** Participants reported that attending the intervention from their own homes via video link was highly convenient Participants reported that the interactive nature facilitated their engagement and gave them the opportunity to talk about their concerns and difficulties. Participants found the length, pacing and time between sessions acceptable. Participants commented that they did not feel overwhelmed by information because the content was pitched at the right level.

. *“It’s so convenient because you don’t have to go anywhere, erm…I think, it was…yeah as I say we’re-we’re time poor, we don’t have to get a sitter or you know…that’s a barrier for us sometimes, erm…it having to go somewhere is even more effort, erm so yeah it was very convenient.” Participant 1429.*

***Changes.*** All participants identified changes which they attributed to the intervention: improved understanding of anxiety and its impact, an increased awareness and ability to recognise signs of anxiety and improved confidence in managing their child’s anxiety. Participants recognised that they would not have felt empowered in this way if their child had attended an intervention with minimal parent involvement, or with no parent involvement at all. Parents found it relatively easy to incorporate the strategies learned during the intervention into their daily lives, and all participants who completed the intervention stated that they would continue to use them in the future.

*“I definitely feel calmer about the situation and when he’s starting up into anxiety that would quite often make me feel (intake of breath) ‘oh no it’s happening again’ you know so now I tend to take a step back from that and go okay and I’ll just give myself a minute and give him a minute, to just get, to get there, err and to have that moment” (Participant 1968).*

All participants who completed the intervention also noticed positive changes related to their child’s mood and behaviour: a reduction in child anxiety and an improved ability to initiate or engage in conversations about fears and worries. *“I’m still waiting for the CAMHS referral so, but I don’t feel like we need it now really”* (*Participant 1586).*

***Connection with Other Parents.*** All participants who attended the intervention with at least one other parent commented on the value of hearing other parents’ experiences and sharing learning with each other, resulting in participants feeling less isolated with the difficulties they were experiencing with their child. Those who were the only participant in the intervention reflected on the potential benefits of learning from other parents in a group environment.

*“It was also really supportive to have those other parents there, in similar situations going through similar experiences, that had such a massive effect on me more than I expected it to because, I found erm, I found it really reassuring to know that I wasn’t alone” (Participant 1397).*

## Theme 2: Challenges Associated with the Intervention

***Barriers.*** The main barriers described by participants related to the online delivery of the intervention via online videoconferencing; participants expressed mixed views. Although participants seemed to value the convenience of being able to attend the intervention from their own homes, some reflected that the demands of family life made it difficult to fully engage in the sessions.

*“I would have liked to have been able to give it my full, 100% concentration…Yeah having the brain capacity, to be able to think about it properly without people running up to me and saying ‘mummy’ or bouncing around or me feeling guilty that they’re on tablets or (laughs)” (Participant 1259).*

Participants described issues with sound; this was due to background noise, or difficulties with Zoom only registering one voice if two people speak simultaneously. Participants also commented on the loss of personal connection with other parents, missing out on a sense of solidarity and the sociable conversations which would occur during a face-to-face group.

*“I think sometimes when you meet people in a group, you’ve got a chance to share more you’ve got a bit more time together, and I think on Zoom as well because you very much, take turns and, whereas, like for example if we were having a break in a group, you’d probably chat amongst yourselves and you’d get to know the parents a little bit more” (Participant 1586).*

Some participants found the wording of items on questionnaires confusing, particularly on the CAPES, which resulted in some missing data.

***Suggested Improvements.*** Participants indicated that they would like more, or longer, sessions in order to facilitate practice of strategies and to provide more time for conversations relating to specific issues. Participants described the potential utility of larger groups, with some suggesting that it would be helpful for both parents to attend the sessions.

Participants also suggested changes to the online delivery, for example, asking participants to stay on mute when not talking, or providing instructions for accessing Zoom. Some participants stated that they would have preferred attending a face-to-face intervention, whilst acknowledging that this was not possible at the time due the restrictions in England resulting from the COVID-19 pandemic (March 2020-April 2021).

### Preliminary Clinical Outcomes

Mean scores, standard deviations and pre-test post-test effect sizes for clinical outcomes are presented in Table 5.

**Table 5:** Mean Scores and Effect Sizes for Clinical Outcomes

	Session 1			Session 2			Session 3				Effect Size
	N	Mean	SD	N	Mean	SD	N	Mean		SD	*d*
PAS	11	62.64	11.46	11	54.18	18.05	11	54.81		16.26	-0.68
SCAS parent	7	34.71	7.83	7	25.14	9.62	7	25.43		10.81	-1.19
SCAS child	5	31.20	15.10	6	32.17	15.28	6	27.83		12.04	-0.22
CAPES behaviour	16	27.56	10.41	16	24.00	12.13	16	24.25	11.98		-0.32
CAPES emotional	16	5.31	1.62	16	4.31	2.08	16	4.13	1.82		-0.73
CAPES Intensity	16	32.87	9.99	16	28.31	13.01	16	28.38	12.86		-0.45
CAPES Efficacy	16	114.43	46.58	16	126.25	51.68	16	133.31	54.47		0.41

ES = effect size, PAS = Preschool Anxiety Scale; SCAS = Spence Child Anxiety Scale, CAPES = *Child Adjustment and Parent Efficacy Scale*

Scores on both parent and child rated outcome measures reflected a reduction in child anxiety from session one to session three. Eight (42.1%) participants showed improvement in terms of reduced T-scores on the PAS or SCAS-parent version. The T-scores of eight (42.1%) participants remained the same and the scores of two (10.5%) participants increased by one point. Three (15.8%) participants obtained a T-score of less than 60 post-intervention. No individual participant scores on the PAS, SCAS-parent and SCAS-child reached the threshold for clinically significant improvement when assessed using the RCI (Jacobson & Truax, 1991).

Calculated effect sizes (Morris, 2008) were moderate and large for parent-rated outcomes (PAS *d* =-0.68; SCAS-parent *d* =-1.19). A small effect size was found when measuring child self-reported anxiety (SCAS-child *d* =-0.22). The effect size for children’s behavioural problems was small (*d* =-0.32) and a moderate to large effect size was found when measuring children’s emotional problems (*d* =-0.73). The effect size for the magnitude of change in total intensity was small to moderate (*d* =-0.45). Parents reported increased confidence/efficacy across all three sessions with a small to moderate effect size (*d* = 0.41).

## Discussion

The aim of the present study was to investigate the feasibility and acceptability of a brief cognitive behavioural group intervention for parents of primary school children experiencing anxiety. The findings suggest that it was feasible to recruit and retain participants in the study. However, only 35 parents expressed interest in the study over the nine-month recruitment period. These recruitment issues may be attributable to the closure of schools due to COVID-19. This required parents to become more involved in their children’s schooling whilst continuing to work, working from home, or potentially being furloughed; parents may not have felt able to participate in an intervention which would create extra demand on their time. One of the two schools initially involved in recruitment was able to continue with recruitment during the pandemic, contacting parents with study information remotely from mid-April 2020 (for example, email and social media posts). However, the other involved school was unable to support recruitment to the study until October 2020. Eight of the 19 participants were recruited from this school. Between July and September 2020, primary schools, children’s centres, out of school clubs, children’s sports clubs, girl guides and scouting groups were contacted in an attempt to continue recruitment. However, it is likely that parents had less contact with these groups during the lockdown, and the leaders of these groups may have found it difficult to prioritise recruitment to the study during the pandemic.

Furthermore, the first four groups were delivered with just one participant and only two of the eight groups were carried out with more than two parents. As such, four participants received an individual rather than a group intervention. The treatment fidelity checklist was closely followed to ensure that deviation from the format and content of the intervention did not occur during these first four groups. Whilst those participants still reported positive parent and child changes, this may have implications for the feasibility of intervention delivery. In this study, using a fixed start date for the intervention may have compromised the delivery of the intervention to larger groups of parents. In line with the aim of maximising retention, it was decided not to keep participants on a waiting list to attend a larger group. However, a future trial could consider increased flexibility, perhaps only carrying out the intervention when at least five participants have been recruited (Biggs et al., 2020) to provide more data regarding the feasibility of delivering this intervention in a group format. It may also be helpful to explore parent motivation and engagement with materials sent from schools and children’s centres to inform recruitment processes for future research.

Almost one third of participants (31.6%) did not complete the intervention, i.e., attended fewer than 2 out of the 3 sessions. The groups were offered and delivered at different times (five in the morning, three in the evening); all attrition in the study resulted from the morning sessions. Participants providing reasons for non-completion cited work commitments as the main barrier to attendance. However, there may be some important differences between those participants who did or did not complete the study, such as socioeconomic status, competing demands or perceived suitability of the intervention. The timing of sessions should be considered to further improve retention rates.

Preliminary clinical outcomes suggested that participants found this to be an acceptable and useful intervention. Whilst participants were satisfied with session length and fortnightly delivery, qualitative data indicated that participants would like more or longer sessions. This finding is in contrast to previous research suggesting that parents would prefer briefer interventions to reduce burden on busy families (Tully & Hunt, 2016).

Participants commented on the value of connecting with other parents, which reduced feelings of isolation. This connection was achieved despite the intervention being delivered online via Zoom, which was identified as a barrier to social interaction by some participants. This feeling of connection was achieved when just two parent participants attended the intervention, which may be a reflection of the isolation and loss of support that parents may have experienced during lockdown. Group interventions can provide the opportunity for parents to receive peer support and validation (Navaneetham & Ravindran, 2017).

Participants described some confusion when completing the CAPES. These difficulties particularly related to items rating parent confidence for managing behaviours that their child did not engage in, or in relation to positive behaviours, which resulted in participants choosing not to complete all of the confidence ratings. Wittkowski et al.’s (2017) systematic review of parent self-efficacy measures identified three alternatives to the CAPES suitable for parents of children aged 4 to 10 years old: Comfort with Parenting Performance (Ballenski & Cook, 1982), Me as a Parent (MaaP; Hamilton et al., 2015) and the Parental Self-Agency Measure (PSAM; Dumka et al., 1996). The CAPES was chosen for the current study due to the inclusion of specific emotional and behavioural items (e.g., ‘rate your confidence: my child seems fearful and scared’) rather than more general statements (e.g., ‘I know I am doing a good job as a parent’; Hamilton et al., 2015). It may be that participants require some additional support or specific instruction regarding the completion of questionnaires.

It is important to consider how this intervention should be offered in the future. Participants valued the convenience of the online delivery of the intervention, which reduced barriers for engagement, such as organising childcare and travel. Our findings suggest that the intervention was successfully adapted to an online format. However, the majority of barriers were related to the online delivery of the intervention. Some of these barriers could potentially be removed by providing participants with clear instructions and information about Zoom in advance. Parents expressed concerns about the original intention to deliver the intervention at their child’s school. Previous literature has highlighted parental concern for negative consequences at school if their child were labelled as anxious, including anxiety being included on their child’s school record, moving to a different class and bullying by peers (Chavira et al., 2017). Interestingly, all participants stated that they would have attended the intervention if delivered in a clinical setting (e.g., CAMHS, GP surgery). This highlights a bias in the current sample as wider literature has highlighted barriers for parents accessing psychological services (Reardon et al., 2017).

### Clinical Outcomes

Intervention attendance appeared to be associated with beneficial change for both parents and children. Effect sizes for parent-rated outcome measures were large. However, a more modest treatment effect was found for child self-reported outcome measures. The reasons for this discrepancy are unclear, despite it being a longstanding feature within the literature (Engel et al., 1994). Participants reported that they did not have the opportunity to use all of the strategies during the six-week-timeframe of the intervention. Therefore, whilst parents received benefits such as validation of their difficulties and increased understanding of anxiety, their child may have had less opportunity to experience change.

Although positive changes were described by participants, only three participants (15.8%) obtained a T-score of less than 60 post-intervention, indicating that anxiety was no longer at a subclinical or elevated level. In the absence of a control group, any reported changes cannot reliably be attributed to the intervention. Approximately half of participants (52.6%) obtained T-scores of ≥ 70 during eligibility screening. Whilst the PAS and SCAS should not be used diagnostically, this could indicate that children were experiencing more severe anxiety than expected in this study. An alternative possibility is that parents and children have different conceptualisations of anxiety (Nauta et al., 2004), or parents over-estimate their child’s level of anxiety. Many of the participants experienced anxiety themselves, and parent beliefs about their child’s experience of anxiety have been found to mediate the link between parent and child anxiety (Francis & Chorpita, 2011). It may be that the intervention was more effective at changing parent perceptions of child anxiety than having an indirect effect upon the child’s experience of anxiety. These findings could also reflect the context of this study which took place during the global COVID-19 pandemic; anxiety and uncertainty were likely to be higher for both parents and children and access to usual coping strategies, such as support from friends and family, were limited due to lockdown restrictions.

Self-report data were not included for children under the age of seven years old. This is due to difficulties in administering questionnaires to young children with limited literacy skills who developmentally are less able to reflect on their own mental state (Luby et al., 2007). However, the majority of parents with children under the age seven years old reported that it would be beneficial for their child to complete a measure of anxiety themselves. It would be valuable to explore alternative methods of capturing young children’s experience of anxiety in future research.

### Strengths and Limitations

A considerable strength of this study was that both quantitative and qualitative data from parents were captured, obtaining richer information on the feasibility and acceptability of the intervention. However, as this study was a small scale, uncontrolled feasibility study, it cannot be concluded that reductions in child anxiety were a direct result of the intervention. Due to the study design and small sample size, effect sizes may be inflated and so the results must be interpreted with caution. As such, clinical outcomes from this study can only be viewed as preliminary.

All participants self-referred into the study; many of the parents experienced anxiety themselves. Participant self-selection into the study may reflect a biased sample of particularly motivated parents which may not be representative of the wider parent population. Almost all participants self-identified as White British, again limiting the generalisability of findings. The majority of participants in the study was mothers. Information on socio-economic status was not collected from participants; however, this data could have provided further information of any economic diversity within the sample and improved generalisability of study results.

Participants from two-parent families indicated that attendance was simply due to availability. Interview data from one father suggested that he was concerned about being the only father, though this did not affect his attendance. Understanding the barriers to father participation and successfully engaging fathers in parent-only interventions could lead to further positive outcomes for children.

The present study relied on self-report data from parents which is subject to response bias, social desirability and misunderstanding or misinterpretation of questionnaire items. Parents of children aged seven years and above were asked to encourage their child to complete the SCAS-child questionnaire. Although they were asked not to intervene with child responses, simply by being asked to complete the questionnaire by their parents could have introduced bias into children’s responses. The first author (CJ) delivered the intervention, conducted interviews, collected and analysed data, which may also have introduced bias in participants’ responses.

### Clinical Implications and Future Research

This study should be considered in the context of the UK’s national focus on early intervention and prevention in children and young people’s mental health (Department of Health & Department for Education, 2017). The results demonstrate that a brief cognitive behavioural group intervention is feasible and acceptable for parents of children experiencing anxiety in the absence of a diagnosed anxiety disorder. This brief intervention could increase accessibility and improve parent engagement in psychological support for their child. This intervention may be particularly useful for parents who may find it difficult to access CAMHS, yet still feel that they need help. Preliminary analysis also indicates that this intervention may have potential clinical benefits in reducing child symptoms of anxiety. This intervention may be a useful waiting list intervention, preparing families for accessing psychological therapy, increasing parent efficacy in managing anxiety, and perhaps in some cases resulting in no further need of services.

Next steps for research should involve the development of a larger pilot trial of this group interventions for parents. This would need to consider feasibility of trial processes including use of a control group, randomisation, blinding and a longer term follow-up, which were not investigated within this initial study. It is important to consider how the intervention should be offered in a future trial to better understand the feasibility of an online intervention beyond the current context, where lockdown restrictions made online delivery the only available option for parents. A future trial could also evaluate the cost-effectiveness of the intervention to provide further data on the feasibility of a group intervention. This trial should recruit a larger, more diverse sample with an aim to include more fathers in the overall sample. A larger, more representative sample could increase the reliability of the clinical outcomes. Inclusion of a long-term follow up of six months would be beneficial in order to identify any long-lasting clinical benefits of the intervention, including whether child-rated outcomes show more change over time as parents have more opportunities to implement strategies.

## Conclusions

This feasibility and acceptability study has demonstrated the potential for a brief cognitive behavioural group intervention, delivered exclusively to parents of children experiencing anxiety. Participant retention rates and satisfaction scores were high. Calculated effect sizes indicated reductions in child anxiety symptoms rated by both parents and children, suggesting that the intervention may have potential clinical benefits. However, a definitive trial with a larger sample size is required to further investigate these preliminary findings.

## References

[CR1] American Psychiatric Association (2013). *Diagnostic and statistical manual of mental disorders* (5th ed.). 10.1176/appi.books.9780890425596

[CR2] Ando, H., Cousins, R., & Young, C. (2014). Achieving Saturation in Thematic Analysis: Development and Refinement of a Codebook. *Comprehensive Psychology*, *3*(4), 10.2466/03.CP.3.4

[CR3] Attkisson CC, Zwick R (1982). The client satisfaction questionnaire: Psychometric properties and correlations with service utilization and psychotherapy outcome. Evaluation and Program Planning.

[CR4] Ballenski C, Cook A (1982). Mothers’ Perceptions of Their Competence in Managing Selected Parenting Tasks. Family Relations.

[CR5] Biggs K, Hind D, Gossage-Worrall R, Sprange K, White D, Wright J, Cooper C (2020). Challenges in the design, planning and implementation of trials evaluating group interventions. Trials.

[CR6] Bittner A, Egger HL, Erkanli A, Jane Costello E, Foley DL, Angold A (2007). What do childhood anxiety disorders predict?. Journal of Child Psychology and Psychiatry.

[CR7] Braun V, Clarke V (2006). Using thematic analysis in psychology. Qualitative Research in Psychology.

[CR8] Cartwright-Hatton S, McNally D, Field AP, Rust S, Laskey B, Dixon C, Woodham A (2011). A new parenting-based group intervention for young anxious children: results of a randomized controlled trial. Journal of the American Academy of Child and Adolescent Psychiatry.

[CR9] Cartwright-Hatton S, Laskey B, Rust S, McNally D (2010). From Timid to Tiger: A Treatment Manual for Parenting the Anxious Child.

[CR10] Chavira DA, Bustos C, Garcia M, Reinosa Segovia F, Baig A, Ng B, Camacho A (2018). Telephone-assisted, parent-mediated CBT for rural Latino youth with anxiety: A feasibility trial. Cultural Diversity & Ethnic Minority Psychology.

[CR11] Chavira DA, Bantados B, Rapp A, Firpo-Perretti YM, Escovar E, Dixon L, Palinkas LA (2017). Parent-reported stigma and child anxiety: A mixed methods research study. Children and Youth Services Review.

[CR12] Chavira DA, Drahota A, Garland AF, Roesch S, Garcia M, Stein MB (2014). Feasibility of two modes of treatment delivery for child anxiety in primary care. Behaviour Research and Therapy.

[CR13] Creswell, C., & Willetts, L. (2007). *Overcoming your child’s fears and worries: a self-help guide using cognitive behavioural techniques*. Constable & Robinson

[CR14] Creswell C, Hentges F, Parkinson M, Sheffield P, Willetts L, Cooper P (2010). Feasibility of guided cognitive behaviour therapy (CBT) self-help for childhood anxiety disorders in primary care. Mental Health in Family Medicine.

[CR15] Dempster R, Wildman B, Keating A (2013). The role of stigma in parental help-seeking for child behavior problems. Journal of Clinical Child and Adolescent Psychology.

[CR16] Department of Health, & Department for Education (2017). Transforming Children and Young People’s Mental Health Provision: a Green Paper. https://assets.publishing.service.gov.uk/government/uploads/system/uploads/attachment_data/file/664855/Transforming_children_and_young_people_s_mental_health_provision.pdf

[CR17] Dumka L, Stoerzinger H, Jackson K, Roosa M (1996). Examination of the Cross-Cultural and Cross-Language Equivalence of the Parenting Self-Agency Measure. Family Relations.

[CR18] Engel NA, Rodrigue JR, Geffken GR (1994). 1994/12/01). Parent-Child Agreement on Ratings of Anxiety in Children. Psychological Reports.

[CR19] Francis SE, Chorpita BF (2011). Parental Beliefs About Child Anxiety as a Mediator of Parent and Child Anxiety. Cognitive Therapy and Research.

[CR20] Golding, K. S., & Hughes, D. A. (2012). *Creating Loving Attachments: Parenting with PACE to Nurture Confidence and Security in the Troubled Child*. Jessica Kingsley Publishers

[CR21] Hamilton VE, Matthews JM, Crawford SB (2015). Development and Preliminary Validation of a Parenting Self-Regulation Scale: “Me as a Parent”. Journal of Child and Family Studies.

[CR22] Jacobson NS, Truax P (1991). Clinical significance: a statistical approach to defining meaningful change in psychotherapy research. Journal of Consulting and Clinincal Psychology.

[CR23] James, A. C., James, G., Cowdrey, F. A., Soler, A., & Choke, A. (2015). Cognitive behavioural therapy for anxiety disorders in children and adolescents. *Cochrane Database of Systematic Reviews, (2).*10.1002/14651858.CD004690.pub4 10.1002/14651858.CD004690.pub4 PMC6491167 25692403

[CR24] Jewell C, Wittkowski A, Pratt D (2022). The impact of parent-only interventions on child anxiety: A systematic review and meta-analysis. Journal of Affective Disorders.

[CR25] Jones T, Prinz R (2005). Potential roles of parental self-efficacy in parent and child adjustment: A review. Clinical Psychology Review.

[CR26] Larsen DL, Attkisson CC, Hargreaves WA, Nguyen TD (1979). Assessment of client/patient satisfaction: Development of a general scale. Evaluation and Program Planning.

[CR27] Lebowitz ER, Omer H, Hermes H, Scahill L (2014). Parent training for childhood anxiety disorders: The SPACE program. Cognitive and Behavioral Practice.

[CR28] Luby JL, Belden A, Sullivan J, Spitznagel E (2007). Preschoolers’ Contribution to their Diagnosis of Depression and Anxiety: Uses and Limitations of Young Child Self-Report of Symptoms. Child Psychiatry and Human Development.

[CR29] Mahoney G, Wiggers B (2007). The Role of Parents in Early Intervention: Implications for Social Work. Children & Schools.

[CR31] Monga S, Rosenbloom BN, Tanha A, Owens M, Young A (2015). Comparison of child-parent and7 parent-only cognitive-behavioral therapy programs for anxious children aged 5 to 7 years: 8 short- and long-term outcomes. Journal of the American Academy of Child and Adolescent Psychiatry.

[CR32] Morawska A, Sanders MR, Haslam D, Filus A, Fletcher R (2014). Child Adjustment and Parent Efficacy Scale: Development and Initial Validation of a Parent Report Measure. Australian Psychologist.

[CR33] Morris SB (2008). Estimating Effect Sizes From Pretest-Posttest-Control Group Designs. Organizational Research Methods.

[CR34] Nail JE, Christofferson J, Ginsburg GS, Drake K, Kendall PC, McCracken JT, Sakolsky D (2015). Academic Impairment and Impact of Treatments Among Youth with Anxiety Disorders. Child & Youth Care Forum.

[CR35] National Institute for Health and Care Excellence (2013). Social anxiety disorder: recognition, assessment and treatment. Clinical guideline [CG159]. http://www.nice.org.uk/guidance/cg159 31869048

[CR36] National Health Service (2017). Next steps on the NHS Five Year Forward View. https://www.england.nhs.uk/wp-content/uploads/2017/03/NEXT-STEPS-ON-THE-NHS-FIVE-YEAR-FORWARD-VIEW.pdf 10.1136/bmj.j1678 28377430

[CR37] Nauta MH, Scholing A, Rapee RM, Abbott M, Spence SH, Waters A (2004). A parent-report measure of children’s anxiety: psychometric properties and comparison with child-report in a clinic and normal sample. Behaviour Research and Therapy.

[CR38] Navaneetham NJ, Ravindran D (2017). Group Work Intervention for the Parents of Children with Mental Health Issues Admitted in the Tertiary Care Center. Indian Journal of Psychological Medicine.

[CR39] NHS Digital (2017). Mental Health of Children and Young People in England. Emotional disorders. https://files.digital.nhs.uk/14/0E2282/MHCYP%202017%20Emotional%20Disorders.pdf

[CR41] QSR International Pty Ltd (2018). *NVivo 12 qualitative data analysis software* (Version 12). [Computer software] https://www.qsrinternational.com/nvivo-qualitative-data-analysis-software/support-services/nvivo-downloads

[CR42] Reardon T, Harvey K, Baranowska M, O’Brien D, Smith L, Creswell C (2017). What do parents perceive are the barriers and facilitators to accessing psychological treatment for mental health problems in children and adolescents? A systematic review of qualitative and quantitative studies. European Child & Adolescent Psychiatry.

[CR43] Settipani CA, Kendall PC (2013). Social functioning in youth with anxiety disorders: association with anxiety severity and outcomes from cognitive-behavioral therapy. Child Psychiatry & Human Development.

[CR44] Shahsavari H, Matourypur P, Ghiyasvandian S, Nejad MRG (2020). Medical Research Council framework for development and evaluation of complex interventions: A comprehensive guidance. Journal of Education and Health Promotion.

[CR45] Spence SH (1998). A measure of anxiety symptoms among children. Behaviour Research and Therapy.

[CR46] Spence SH, Rapee R, McDonald C, Ingram M (2001). The structure of anxiety symptoms among preschoolers. Behaviour Research and Therapy.

[CR47] Towe-Goodman NR, Franz L, Copeland W, Angold A, Egger H (2014). Perceived family impact of preschool anxiety disorders. Journal of the American Academy of Child and Adolescent Psychiatry.

[CR48] Tully LA, Hunt C (2016). Brief Parenting Interventions for Children at Risk of Externalizing Behavior Problems: A Systematic Review. Journal of Child and Family Studies.

[CR50] Wittkowski A, Garrett C, Calam R, Weisberg D (2017). Self-report measures of parental self-efficacy: A systematic review of the current literature. Journal of Child and Family Studies.

[CR51] Yalom ID, Leszcz M (2005). The theory and practice of group psychotherapy.

[CR52] Yin B, Teng T, Tong L, Li X, Fan L, Zhou X, Xie P (2021). Efficacy and acceptability of parent-only group cognitive behavioral intervention for treatment of anxiety disorder in children and adolescents: a meta-analysis of randomized controlled trials. Bmc Psychiatry.

